# A generalizable normative deep autoencoder for brain morphological anomaly detection: application to the multi-site StratiBip dataset on bipolar disorder in an external validation framework

**DOI:** 10.1016/j.artmed.2024.103063

**Published:** 2025-01-02

**Authors:** Inês Won Sampaio, Emma Tassi, Marcella Bellani, Francesco Benedetti, Igor Nenadić, Mary L. Phillips, Fabrizio Piras, Lakshmi Yatham, Anna Maria Bianchi, Paolo Brambilla, Eleonora Maggioni

**Affiliations:** a Department of Electronics, Information and Bioengineering, Politecnico di Milano, Milan, Italy; b Department of Neurosciences and Mental Health, Fondazione IRCCS Ca’ Granda Ospedale Maggiore Policlinico, Milan, Italy; c Department of Neurosciences, Biomedicine and Movement Sciences, Section of Psychiatry, University of Verona, Verona, Italy; d Division of Neuroscience, Unit of Psychiatry and Clinical Psychobiology, IRCCS Ospedale San Raffaele, Milan, Italy; e Cognitive Neuropsychiatry Lab, Department of Psychiatry and Psychotherapy, Philipps-University Marburg, Marburg, Germany; f Department of Psychiatry, University of Pittsburgh School of Medicine, Pittsburgh, PA, USA; g Fondazione IRCCS Santa Lucia, Roma, Italy; h Department of Psychiatry, University of British Columbia, Vancouver, BC, Canada; i Department of Pathophysiology and Transplantation, University of Milan, Milan, Italy

**Keywords:** Normative modelling, Anomaly detection, Multi-site harmonization, Psychiatric disorders, Brain MRI

## Abstract

The heterogeneity of psychiatric disorders makes researching disorder-specific neurobiological markers an ill-posed problem. Here, we face the need for disease stratification models by presenting a generalizable multivariate normative modelling framework for characterizing brain morphology, applied to bipolar disorder (BD). We used deep autoencoders in an anomaly detection framework, combined for the first time with a confounder removal step that integrates training and external validation.

The model was trained with healthy control (HC) data from the human connectome project and applied to multi-site external data of HC and BD individuals. We found that brain deviating scores were greater, more heterogeneous, and with increased extreme values in the BD group, with volumes prominently from the basal ganglia, hippocampus, and adjacent regions emerging as significantly deviating. Similarly, individual brain deviating maps based on modified z scores expressed higher abnormalities occurrences, but their overall spatial overlap was lower compared to HCs.

Our generalizable framework enabled the identification of brain deviating patterns differing between the subject and the group levels, a step forward towards the development of more effective and personalized clinical decision support systems and patient stratification in psychiatry.

## 1. Introduction

Psychiatric disorders, as described in the current categorical classification system, are highly heterogeneous marked by a complex interplay of genetic and environmental factors that lead to altered physiological mechanisms [1-3]. Many neuroimaging studies have sought to objectively characterize these disorders by searching for brain markers that could support diagnosis or disease management [4-10]. However, no clinically useful markers have emerged to date [11]. For instance, brain models of bipolar disorder (BD) are currently being investigated, but the overall findings appear fragmented [12,13]. A recurrent challenge lies in the inability to generalize findings from a patient population to the group level, as group-level diagnostic effects have been shown to not replicate at the subject level [14] and appear to be shared between different diagnostic groups [15-17]. This phenomenon is attributed to the fact that the current diagnostic categorization of psychiatric disorders was not informed by neurobiological evidence [18,19]. As a result, delineating disorder-specific neurobiological patterns is challenging and the study of brain morphological markers of psychiatric disorders should account for the uncertainty associated with the diagnostic labels, moving away from classic case-control group comparisons to personalized normative-based statistical inferences [20-22].

Deep learning (DL) autoencoder (AE) models have been widely employed in anomaly detection frameworks and have emerged as suitable multivariate models for brain normative frameworks [23-25]. These models, which are based on artificial neural networks, have an encoder-decoder architecture designed to capture relevant regularities in data through the minimization of the input reconstruction error (RE). The REs are fully traceable, thus enabling the identification of specific brain regions that exhibit higher deviations from the norm. This effectively attenuates the lack of interpretability associated with these model’s “black box” nature.

Leveraging these promising modelling tools and a large multi-site T1-weighted structural magnetic resonance imaging (sMRI) dataset of healthy controls (HC) and individuals with BD, our study proposes a robust and innovative personalized medicine framework for improving the complex clinical management of BD (and other mental disorders). A shift from a disease-centred to a patient-centred paradigm is promoted via the development of a generalizable, and extendable AE-based brain normative modelling and anomaly detection framework. In addition, model interpretability is enhanced through the application of the AE model on confounder-free data [26].

We propose a normative model that integrates multivariate region-based brain morphological data: cortical thickness (CT), gray matter (GMV), and white matter volumes (WMV) features, and, for the first time, a confounders’ removal processing step fully generalizable to external datasets.

An innovative end-to-end pipeline was designed to manage both biological and site-related confounding sources, embedded in an external validation (EV) framework [27]. The normative model framework was trained on the confounders-free brain features extracted from the human connectome project young adults (HCP-YA) dataset [28] and applied to multi-site data from the StratiBip network [16], including HC and subjects with BD. Subject-level REs were compared between HC and BD to assess and characterize deviating brain patterns in affected individuals, under the assumption that patients would express higher normative deviations than HCs, in well-localized brain regions. We hypothesized that the AE-based normative model would be a robust and effective tool to characterize this heterogeneous disorder and identify disentangled subject- and group-level patterns of neuroanatomical alterations.

## 2. Related work

Numerous techniques have been proposed for brain normative modelling and anomaly detection, which we distinguish here as regression-based and DL-based. Unsupervised deep learning models for anomaly detection are mostly based on AEs or Generative Adversarial Networks [25]. According to a recent review, most DL-based anomaly detection techniques for brain medical imaging have been developed for lesion and tumor detection or for brain segmentation, taking raw images and volumes as input [29]. Few examples can be found in literature applying this framework to psychiatric disorders, where brain alterations are subtle and not explicitly present. The first to develop such an application with deep AEs was *Pinaya* et al. [23], training an AE model with brain morphological features from healthy controls and then employing an anomaly detection framework to study brain normative deviations of schizophrenic and autistic patients. In the same line, based on an adversarial AE model, the same author studied brain morphological deviations from patients with Alzheimer’s disease and mild cognitive impairment [24]. More recently, a basic autoencoder was employed as a normative data-driven feature learner and applied to extract data-driven brain-deviating scores [30]. In the latter work, the AE was trained with brain volumetric features from healthy controls and then the test set reconstruction errors associated with controls and subjects affected by bipolar disorder were extracted and fed to a feature selection module and a random forest classifier. Besides the described studies, most normative modelling approaches developed for psychiatric disorders have applied regression methods. In this case, normative brain curves have been mapped mainly using Gaussian process regression (GPR), first proposed for normative modelling in [31], and since then has been extensively used [14,20]. Differently, *C. J. Fraza* et al. [32] proposed warped Bayesian linear regression as an improvement upon the latter GPR, which was successfully implemented in the work developed by *S. Rutherford* et al. [22]. Other regression-based methods have also been proposed, such as generalized additive models [33,34]; nevertheless, all these methods are univariate, since they fit a separate regression line to each brain region and therefore do not address the interdependences among brain regions [35]. Conversely, multivariate approaches can overcome this issue by facilitating the study of pattern-wise brain changes [36]. In *R. Ge* et al. [37], a comparative analysis of eight algorithms, including the aforementioned methods, identified multivariate fractional polynomials (MFP) as the most effective model; still, deep learning models surpass MFPs in capacity and in handling highly complex multivariate relationships.

In summary, the majority of anomaly detection techniques developed for studying psychiatric disorders have relied on regression methods, which are limited in their capacity to model complex multivariate relationships. Few studies have employed DL-based techniques to investigate brain morphological anomalies, and those that have, have not addressed a critical challenge in psychiatry: the heterogeneity of diagnostic groups. The present study aims to fill this gap by proposing an end-to-end normative framework based on deep-AEs and statistical inference methods to study both within-group heterogeneity and between-group discrimination.

## 3. Materials and methods

**Fig. 1.**
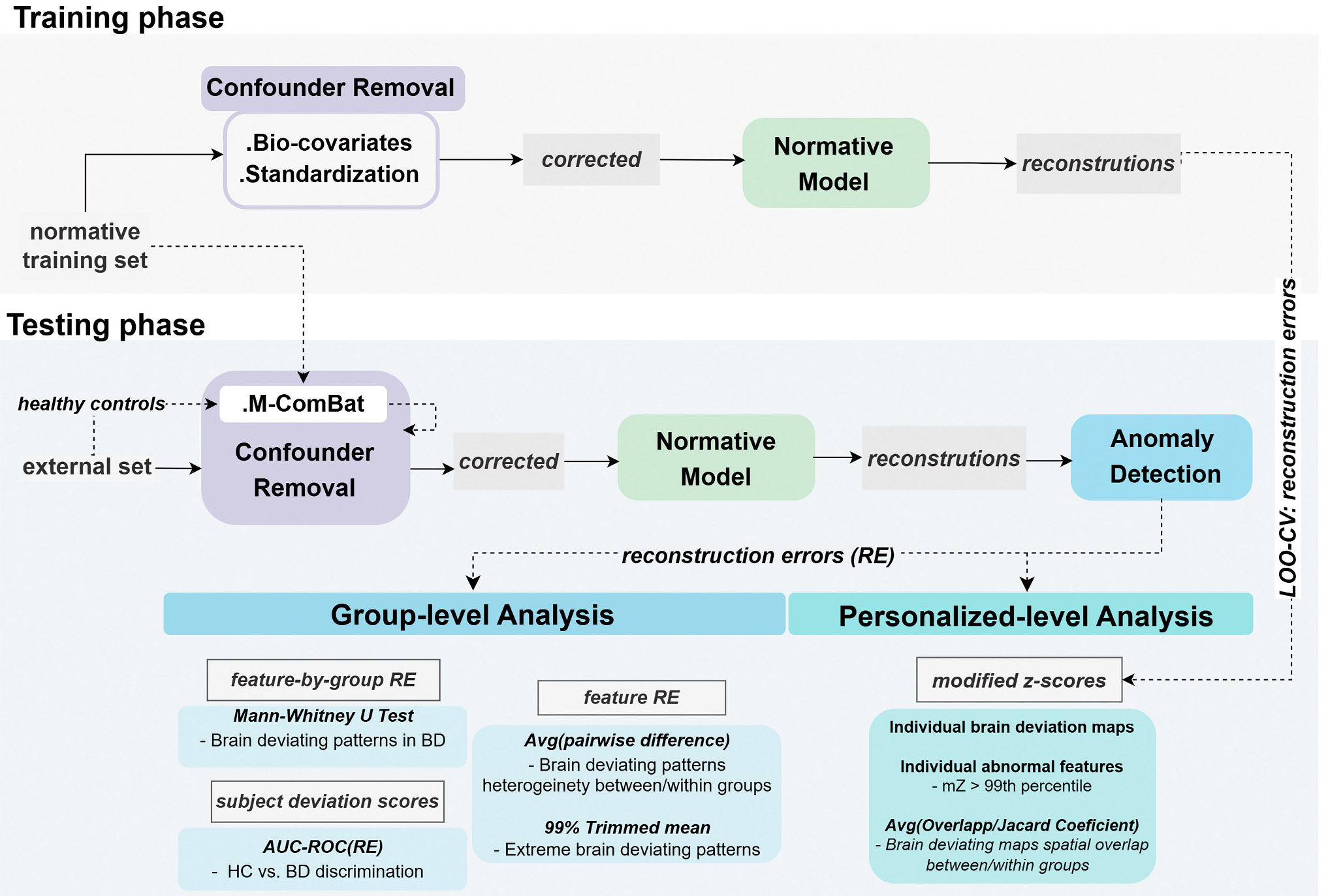
Normative modelling framework. In the training phase, the normative training set is used to fit the biocovariates regression pipeline, and after data correction, the normative AE model is trained. In the testing phase, the external test sets are harmonized with the reference normative training set. Then, the corrected test data is fed to the trained normative model and an anomaly detection framework is applied to the model test reconstructions. Leveraging the REs, group-level and subject-level analyses are conducted to characterize both groups in terms of brain normative deviations.

The data analysis workflow is schematized in Fig. 1. We extracted brain regional features from sMRI data which were fed into an embedded confounder removal (CR) pipeline. This pipeline integrated training with the external test set, accounting for both biological and site confounding effects removal. Then, the normative AE model was trained with the confounder-free HCP-YA training set features. The StratiBip test set REs were extracted from the normative model, and both subject’s and brain features-by-group mean deviation scores (MDS) were calculated employing the *mean square error*. In the group-level analysis, we evaluated the MDS group’s discriminative power, identified significant deviating neuroanatomical patterns in the BD group, and characterized both groups in terms of RE heterogeneity and extreme deviating values. Finally, we constructed personalized subject-level brain deviating maps for all test subjects via modified z scores (mZ) transformation and studied individual abnormalities and groups’ spatial maps overlap.

### 3.1. Data

#### 3.1.1. Normative training set: HCP dataset

Our training set was obtained from the HCP-YA public dataset, *1200 Subjects Data Release (S12000 Release, March 2017)* [28], available on the connectomeDB platform (https://db.humanconnectome.org) [38]. The retrieved data consisted of 3 T T1-weighted sMRI scans from 1109 HC subjects aged between 22 and 37 years (*median age = 29.00 years, 604 females, 505 males*). For this dataset, we obtained the *Restricted Data Access Authorization* by signing and agreeing to the WU-Minn HCP Terms. All methods developed and publication of source codes comply with the obligations and regulations of those terms.

#### 3.1.2. StratiBip dataset: external test set

The external test set consisted of data collected as part of the StratiBip network, an initiative that originated from the ENPACT network [16]. The StratiBip dataset results from the post-hoc integration of multi-site clinical and neuroimaging data collected from HC and subjects with BD, more details can be found in supplementary information.

The sMRI data used as external test set was acquired from 550 subjects, 363 HC *(median age = 27.00 years, 189 females, 174 males)* and 187 with BD *(median age = 30.00 years, 101 females, 86 males)* across 7 sites using T1-weighted sequences on 3 T MRI scanners. Each site employed its own resources, protocols, and sequences (Table S12). Consistent with the HCP training sample, only young adults were included, from 22 to 37 years old (Table S1-S2, and Fig. S1).

#### 3.1.3. Comparing both datasets

A Kruskal-Wallis test revealed significant age differences among the three groups, HCP-YA, StratiBip-HC, and StratiBip-BD (*χ*^*2*^(2) = 34.85; *p < 10^−7^*); the post-hoc comparisons showed that StratiBip-HC were younger than HCP-YA and StratiBip-BD subjects (Table S1). On the other hand, based on a Chi-Square test of independence, no significant differences among the three groups were found for sex proportions *(χ2(2, N = 1659) = 0.6338, p = 0.728)*. More detailed information on the sample characteristics in each site can be found in Table S2.

### 3.2. sMRI pre-processing

All sMRI data were pre-processed in Matlab R2018a (The Mathworks, Inc.^®^) environment. Firstly, T1-weighted images underwent a visual quality check and were converted from DICOM to NIFTI format. Following, the pre-processing was performed using the statistical parametric mapping software (SPM12) version 7771 [39], available at (http://www.fil.ion.ucl.ac.uk/spm/software/spm12/), and the computational anatomy toolbox add-on (CAT12) version 12.7 [40]. The detailed pre-processing pipeline is described in supplementary information. The pre-processed volume-based images were used to extract global measures as total intracranial volumes (TIV), regional cortical thickness measures for the Desikan-Killiany-Tourville (DK40) cortical atlas map [41], consisting of 68 ROIs (Table S13) and regional tissue volumes for the CoBra volume atlas map [42], provided by the Computational Brain Anatomy Laboratory at the Douglas Institute (CoBra Lab). The inclusion of volumetric measures was based entirely on the fully automated CAT12 processing pipeline. Therefore, all CAT12 volumetric estimations (WMV and GMV) using the CoBra atlas were included without any selection based on prior knowledge. CAT12 estimates WMV for GM regions and vice versa, using subject-specific tissue probability maps. WMV estimates for GM regions were interpreted as volume estimations for WM areas adjacent to the specific regions, and vice versa. A total of 50 GMV estimations (Table S14) and 52 WMV estimations (Table S15) were considered. The resulting GMV, WMV, and CT features were subject to the following processing steps.

### 3.3. Confounder removal pipeline

#### 3.3.1. Multi-site M-ComBat harmonization

In this study, we present a framework for the harmonization of external test sets, i.e., data collected in sites differing from the training set. Site effects, arising from inter-site differences in MRI scanners and acquisition protocols, encode latent information that makes data not directly comparable, mask biological effects of interest, and, most importantly, are easily learnable for ML models, potentially confounding these analyses [43]. To address this, we developed a pipeline to harmonize brain features from the multi-site StratiBip external test set with the HCP-YA training set. This step was aimed to remove both intra-test set and inter-dataset differences, enabling the reliable application of the trained AE normative model in an EV framework. The pipeline was based on the ComBat (Combatting Batch Effects) tool, described below.

##### ComBat model.

ComBat [44] is a harmonization method widely employed for neuroimaging datasets and particularly robust for small sample sizes [45,46]. It uses an empirical Bayes (EB) framework to estimate model parameters for each included site, assuming both additive and multiplicative site effects on data, $\gamma_{iv}$, $\delta_{iv}$, for the $i^{th}$ site, $j^{th}$ subject, and $v^{th}$ feature y:

(1) $$y_{ijv}=a_{v}+X_{j}^{T}\beta_{v}+\gamma_{iv}+\delta_{iv}\epsilon_{ijv}$$

Furthermore, it allows for the preservation of subject-specific biological covariates, $X_{j}$. The two site effect parameters are estimated from the standardized biocovariates-free data and then used to adjust the original data, as shown in Eq. 2:

(2) $${\hat{y}}_{ijv}^{ComBat}=\frac{y_{ijv}-{\hat{a}}_{v}-X_{j}{\hat{\beta }}_{v}-\gamma_{iv}^{*}}{\delta_{iv}^{*}}+{\hat{\alpha }}_{v}+X_{j}{\hat{\beta }}_{v}$$

In the original ComBat model, the adjusted data is transformed to a location and scale related to the overall mean and pooled variance of the estimated site effects. Hence, to harmonize data, ComBat depends on the sites available at the moment of estimation, enabling its application exclusively in internal validation frameworks [47-49]. This issue is overcome in M-ComBat which gives the possibility to shift samples to a pre-determined reference batch location, $i=ref\;:{\hat{\alpha }}_{i=ref,v}\;{\hat{\sigma }}_{i=ref,v}$, which we have employed for ML-EV frameworks as done before in [50,76].

##### Harmonization pipeline.

**Fig. 2.**
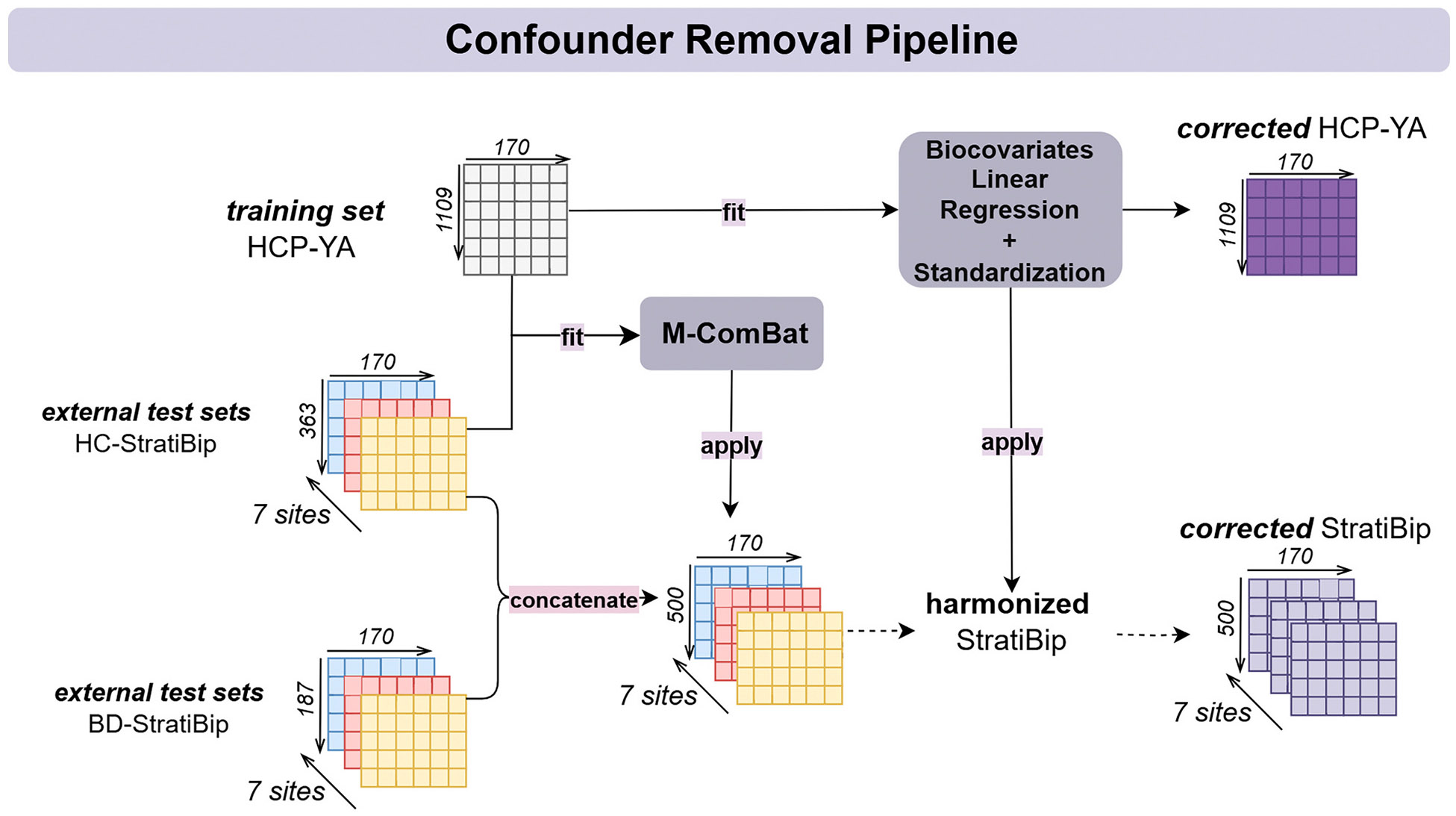
Confounders removal pipeline: M-ComBat harmonization and biocovariates linear regression (LR). The HCP-YA training set is taken as a homogeneous dataset and is only corrected for biological confounders. The external test set is first split into healthy and non-healthy portions. The healthy portion is used to estimate site-effects with the reference normative training set and then the whole test set is harmonized by applying the respective estimated site corrections. Finally, the test data is corrected for biological confounders with the previously fitted regression model and standardized.

We propose an innovative pipeline to enable site harmonization of non-normative external datasets with the normative training set, as shown in Fig. 2. In this case, the M-ComBat model was fitted exclusively on a normative sample and later applied to all test data. First, the external test set (StratiBip) HC portion (*N* = 363), $y_{ij=HC,v}$, is concatenated with the HCP-YA normative training set, which was used as the reference i=HCP to estimate the StratiBip site-effects. In the site-effect estimation stage, the model starts by standardizing data with the HCP-YA statistics ${\hat{a}}_{i=HCP,v}$, ${\hat{\sigma }}_{i=HCP,v}$, while accounting for biological covariates at net of site for all included subjects $y_{ij=HC,v}^{Standardized}=\frac{y_{ij=HC,v}-{\hat{a}}_{i=HCP,v}-X_{ij=HC},{\hat{\beta }}_{v}}{{\hat{\sigma }}_{i=HCP,v}}$. Next, additive and multiplicative site effects were estimated using the EB framework and then applied in the correction stage to harmonize the StratiBip external test set (relative to both HC and BD). The harmonization of the test set was performed as indicated in Eq. 2, using the feature mean, standard deviation, and biocovariates coefficients computed on the HCP-YA reference set.

The python-based *neurocombat* functions made available in (https://github.com/Jfortin1/neuroCombat) by F.P. Fortin were adapted and integrated into a python class available in (https://github.com/inesws/neurocombat_pyClasse), denominated *neurocombat_pyClasse,* compatible with *sklearn Pipelines* and with *fit()*, *transform()* methods for its application in cross-validation (CV) frameworks.

##### Feature harmonization.

Using the pipeline described above, we harmonized TIV, WMV, GMV, and CT features of the StratiBip test set with the reference HCP-YA training set. For all feature sets, age and sex were included as biocovariates to preserve, while for volume features, previously harmonized TIV was also included. First, raw TIV measures were harmonized together with other extracted global measures. Then, regional volumes and CT features were separately harmonized. More detailed information is available in supplementary information.

##### Harmonization pipeline validation.

To ascertain the harmonization success, we proceed with a series of validation analyses. The compliance with the following criteria was assessed: 1) successful and efficacy of site effects removal, 2) total preservation of biological covariates after M-ComBat harmonization. To evaluate 1) we checked if site differences and effects identified before data harmonization were effectively removed after M-ComBat application. We employed Kruskal Wallis ANOVA tests to compare mean feature-type distributions among sites and a site classification paradigm with a support vector machine learning model, before and after harmonization. To evaluate 2) we study the significance of age, sex, and diagnosis effects on raw and harmonized data with linear regression models to assert their stability after M-ComBat harmonization. A more detailed description and complementary analyses are available in supplementary information.

#### 3.3.2. Biological covariates removal via linear regression

After data harmonization, we proceeded with the removal of variance associated with age and sex biocovariates from regional volumes and CT features, and harmonized TIV from volume features, via standard LR [51,52]. We considered the outlined biological covariates as confounding variables as these are implicitly encoded in neuroimaging data and would contribute to a source ambiguity problem of the later developed AE model. We embedded the LR estimations and corrections in the EV, consistently with the proposed CR pipeline. The LR coefficient estimations were performed exclusively on the HCP-YA training set, and the estimated effects were removed from both HCP-YA training set and StratiBip test set [53,54]. After this step, data is referred to as *corrected*. More detailed information can be found in supplementary information.

### 3.4. Autoencoder normative model

After data has been adjusted for the identified confounders, the following step is the implementation of the normative AE-based model.

#### 3.4.1. AE for normative modelling

The implementation of AEs for normative modelling is within the scope of methods for normality feature learning by characterizing regular feature patterns [25]. An AE has an encoder-decoder architecture based on artificial neural networks and is widely used for data embedding representation learning. In the normative framework, the model is trained in an unsupervised fashion to learn to represent normative data by optimizing a generic objective function that minimizes the model reconstruction error. Then, employing an anomaly detection framework, anomalous data instances can be identified by quantifying the reconstruction errors. The working hypothesis revolves around the assumption that *normal* instances can be better reconstructed from the latent space than *anomalous* ones, a difference that can be characterized a posteriori quantifying the reconstruction error.

The structure of AE models follows the following definition: a set of input data, denoted as $X=(x_{1},\ldots ,x_{n})$ is fed to the model. The latent variables, Z, are outputted by an encoder, F(X), and inputted in the decoder G(Z), which is trained to reconstruct X, $\hat{x}=G(z)$. The AE objective is then composed of one term, an unsupervised reconstruction loss [55]:

$${\text{Loss}}_{\text{AE}}=\frac{1}{N}\sum_{j=1}^{N}L_{r}(G(F(x_{j})\;)\;,x_{j})$$

where N denotes samples, and $L_{r}$ the reconstruction loss

#### 3.4.2. Normative model development

We concatenated the three types of brain features - 68 CT, 50 GMV and 52 WMV - into a unified set of 170 features, which was fed as input to the AE model. The AE was designed with a single objective function to guide the reconstruction of the combined dataset, rather than employing separate objective functions for each input data type. This design was aimed at forcing the network to reduce information redundancies encoded into the three modalities and learn domain-relevant interactions by encoding them into a fused compressed latent representation.

The model’s general initial architecture and hyperparameter search space were based on [23]. The model used *selu* activation function and *lecun_normal* weight initialization in all layers [56], except for the last layer of the network that was defined using a *linear* activation function and *gorot* weight initializer. An *l2 norm* was included in all layers for regularization. The model optimization was based on *Adam* [57] and the loss function, $L_{r}$, on the *mean squared error*, $MSE=\frac{1}{N}\sum_{j=1}^{N}{\left(x_{j}-{\hat{x}}_{j}\right)}^{2}$. The number of layers, the number of neurons, the batch size, the number of epochs, the learning rate and the *l2 norm* coefficient were optimized in a 10 fold CV hyperparameter tunning process with a random search strategy as detailed in the supplementary information. After hyperparameter tunning, the best AE model was re-trained on the entire HCP-YA training set.

The model and experiments were implemented with Keras version 3.3.3 and Tensorflow version 2.16.1. The experiments were conducted on a computational platform with a CPU Intel^®^ Core^™^ i7-10700KF CPU @ 3.80GHz 3.79 GHz (32 GB of RAM).

### 3.5. Anomaly detection framework: normative model application

We applied the trained normative AE model to the external StratiBip test set. From the reconstructed StratiBip data, for each feature, we extracted the RE, the squared error between the original and reconstructed instances, $RE_{vj}={\left(x_{vj}-{\hat{x}}_{vj}\right)}^{2}$. Then, the RE values were integrated with the MSE for computing the subject’s mean deviation scores (MDS) by averaging the squared error across all the features: $MDS_{J}=\frac{\sum_{v}^{V}{\left(x_{vj}-{\hat{v}}_{vj}\right)}^{2}}{V}$. To assess model robustness and variability to training data we employed a bootstrap with replacement strategy. The HCP training set underwent a random selection with replacement for 1000 iterations. Each time, an AE normative model was trained with each bootstrap sample and applied to the StratiBip test set. The MDS values resulting from the 1000 bootstraps were subject to the analyses described in the group-level analysis section – BD-deviating brain features. We computed the percentile 95 % confidence intervals (CI) in order to evaluate the variability of model performance and extract statistically significant deviating group-level features, in the BD group.

#### 3.5.1. Group-level analysis

**Fig. 3.**
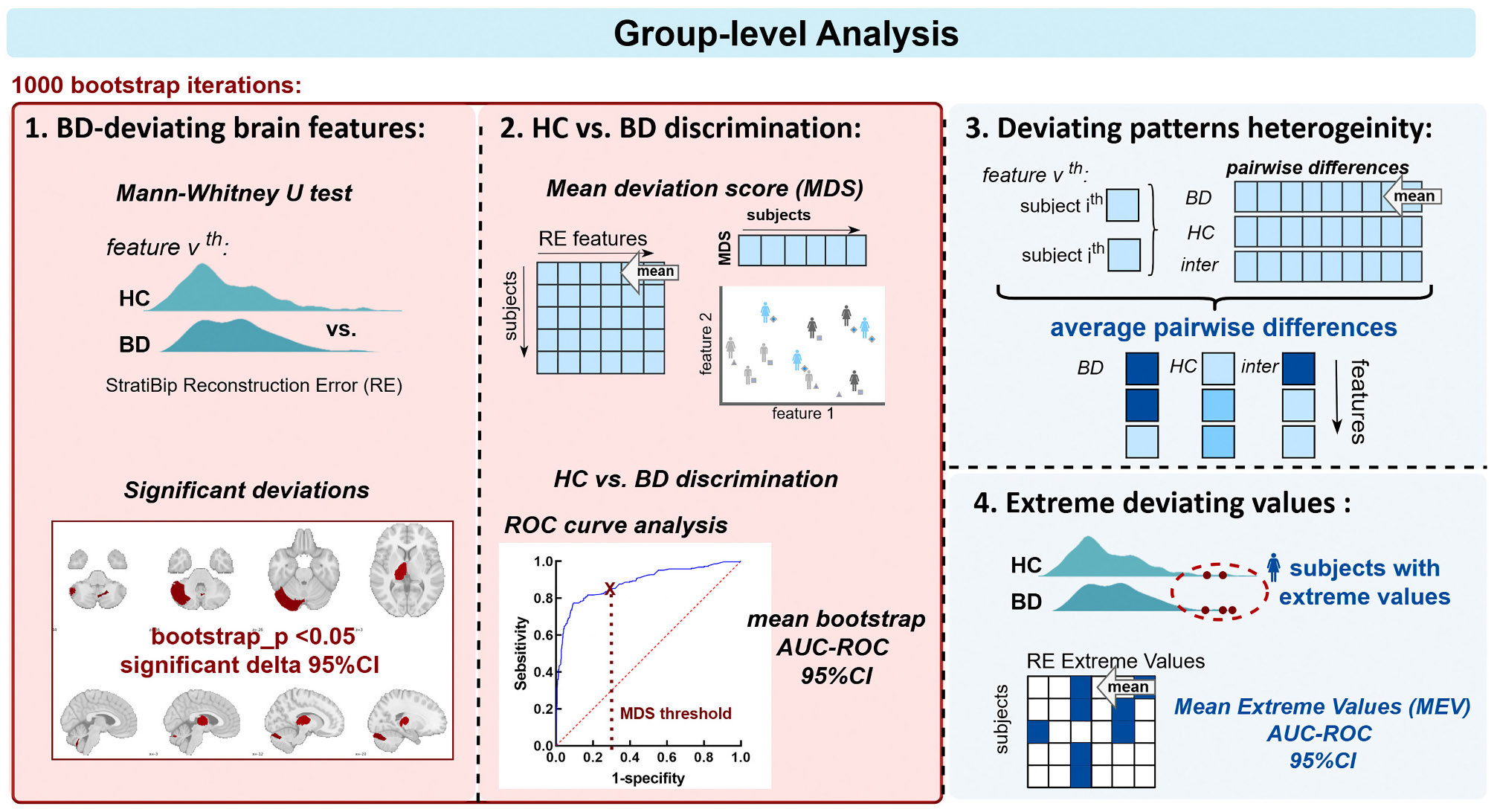
Anomaly detection at group-level. This scheme depicts the four group-level analyses employed to inspect within-group and between-group deviation patterns.

The RE metrics (both RE and MDS) extracted from HC and BD individuals of the StratiBip test set were entered in the following group comparisons, as illustrated in Fig. 3.

##### 3.5.1.1. BD-deviating brain features. AE-based anomaly detection.

To assess whether BD individuals differed from HC in terms of their deviation outcomes from the AE normative model, group-level BD vs. HC comparison of feature-RE values was performed.

First, the median MDS between HC and BD subjects were compared. Then, feature-specific RE distributions were compared to identify region-based brain deviating patterns at the group level. For each brain morphological feature, we compared the RE non-normal distributions between HC and BD, using a one-tailed Mann-Whitney U (MWU) test (alternative hypothesis: BD group median to be higher than the HC group), assigning a critical level of 0.05 (uncorrected), and computing the cliff’s delta effect size to quantify the magnitude of the differences. The initial significance criterion was established by evaluating the *p-value* 95 % CI, accepting all tests with a mean p-value bootstrap estimate of <0.05 (Fig. 3.1). For the features identified from this initial assessment, the effect size was subsequently evaluated and considered significant if its 95 % CI excluded zero [24]. The features resulting from this second-level assessment were identified as having significant increase deviations in the BD group.

###### Mass-univariate analysis.

We performed a standard mass-univariate analysis to facilitate the interpretation of findings regarding the BD normative deviating brain features results from the previous section. Consistently with our pipeline, the corrected features used in this analysis were the same fed to the AE-normative model. A two-tailed MWU test mass-univariate analysis was employed to assess differences between the distributions of the original corrected feature sets between BD and HC group. The critical level was set to 0.05 and a Bejamini-Hochenberg false discovery rate (FDR) correction was employed for multiple comparisons.

###### MDS-based discrimination of BD vs. HC: ROC curve analysis.

Following, we evaluated whether the resulting brain deviations, quantified through the MDS, could discriminate the two StratiBip groups. Each subjects’ REs was summarized with the MDS and a receiver operating characteristic (ROC) curve analysis was employed. The area under the curve (AUC) of the ROC curve was extracted and the optimal discriminative MDS threshold was identified (Fig. 3.2).

##### 3.5.1.2. RE patterns heterogeneity.

After assessing group differences we investigated RE patterns heterogeneity within and between groups (Fig. 3.3). We computed the pairwise feature RE absolute differences between every two subjects, in each group separately and then between groups. Then, we summarized the overall results feature-wised with the mean heterogeneity, Eq. 4, where v stands for feature, j1 and j2 denote two subjects from the same group with N total subjects, and m a selected subject from a different group with M total subjects. The more the RE outcomes varied across subjects for a specific brain feature, the higher the average difference and the heterogeneity.


(4) $$\text{Within Group}\;:\frac{\sum_{j1}^{N-1}\sum_{j2=j1+1}^{N}{\text{RE}}_{j1,v}-{\text{RE}}_{j2,v}}{\frac{1}{2}N(N-1)}\;\text{Between Group}\;:\frac{\sum_{j}^{N-1}\sum_{m}^{N}{\text{RE}}_{j,v}-{\text{RE}}_{m,v}}{\left(M-1)(N-1\right)}$$

##### 3.5.1.3. RE extreme deviations.

Afterward, we moved away from the description of group central tendencies, i.e., comparing medians/mean, and exploited extreme value statistics concepts to investigate the profiles of the RE distribution tails (Fig. 3.4). First, a leave-one-out (LOO)-CV was performed to extract unbiased reconstructions for all HCP-YA training set subjects. In each fold, all subjects except one were used to train the normative AE model. The left-out subject was used as test sample and its reconstruction was extracted. Then, we applied a block maxima approach, where a series of independent observations are summarized by its maximum value within a specific block [58]. In our case, in each group, each feature was considered a block of data with N independent subjects’ measurements and was summarized with the top 1 % mean of extreme values (MEV), i.e., the 99 % trimmed mean, Eq. 5, where k is the number of data points corresponding to the top 1 %. We assessed differences in terms of MEVs for each feature in the three groups, StratiBip HC and BD, and HCP-YA.


(5) $$MEV_{j=group,v}=\frac{1}{k}\sum_{i}^{k}RE_{i,v}$$

#### 3.5.2. Personalized brain deviating maps

**Fig. 4.**
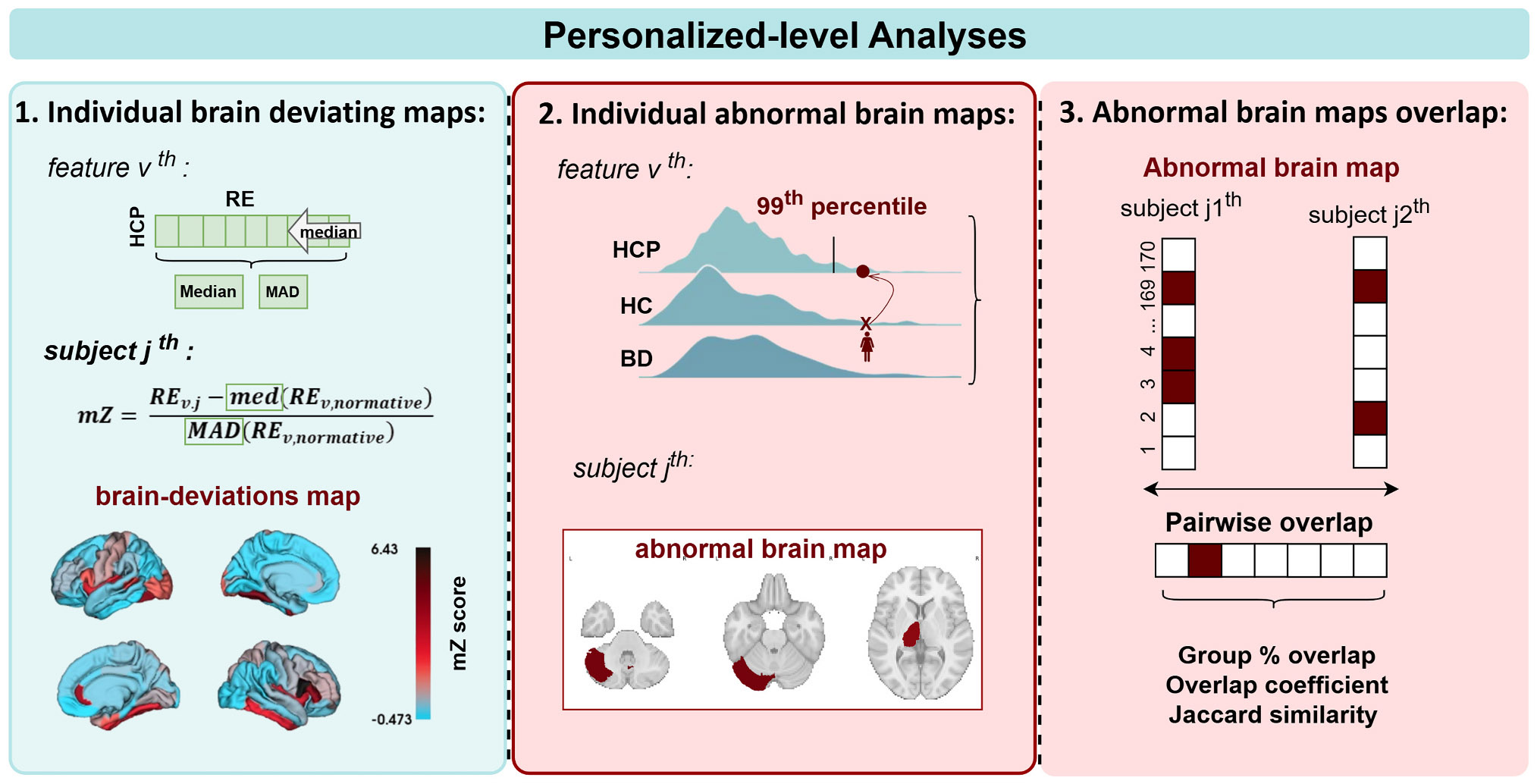
Anomaly detection at personalized-level. This scheme depicts the personalized-level analyses, including the extraction of individual brain deviating maps, the computation of the abnormal maps and the spatial overlap comparisons.

The normative training set and external test set REs were used to compute the mZ scores and derive the individual brain deviating maps and binarized abnormal maps, as shown in Fig. 4.

##### 3.5.2.1. Modified z scores.

The most promising application of the proposed AE normative modelling framework is to move from group-level to individualized analyses. We charted the StratiBip test set features REs by comparing them with the distributions extracted from the HCP-YA training set with the LOO-CV analysis, via modified z scores (mZ), (Fig. 4.1). The mZ scores account for the median and median absolute deviation (MAD) and is more robust than its parametric version for outlier identification when the underlying data distribution is non-normal [59]. Besides, MAD is a robust measure that captures the dispersion around the median while not being influenced by extreme values and the range of the dataset. First, analysing the HCP-YA normative RE outcomes, we calculated the RE median for each feature, $E[RE_{HCP,v}]$, which we considered as the expected model normative RE. Then, we calculated the MAD, the measure of model uncertainty for reconstructing feature v, adjusting the MAD with a correction factor of 1∕Q(0.75), where Q(0.75) corresponds to the 75th quantile in the respective normative feature distribution [59]. Then, the mZ score foresees that each new data point be standardized with the median and MAD of the normative expected RE distribution, Eq. 6, and was used to compute personalized deviating brain maps for each subject in the StratiBip test set. Afterward, we defined an abnormality criterion based on the MAD, to derive abnormal features at the individual level (Fig. 4.2). Usually, when data is normally distributed, a known threshold for outlier detection is the measure of 3 standard deviations, or 3.5 MADs [60,61]. In our case, we defined a threshold for each feature based on its specific normative RE distribution. Our data did not follow a normal distribution and we assume that each feature was encoded differently by the model, having different expected normative RE outcomes. Thus, we translated this feature-specific encoding into a definition of feature-specific abnormal thresholds. For each normative RE feature distribution, we took the mZ threshold corresponding to the 99th percentile. Thus, an individual feature was considered abnormal if fell in the top 1 % of the normative RE expected distribution.


(6) $$mZ_{jv}=\frac{RE_{jv}-E[RE_{HCP;v}]}{MAD_{HCP;v}}$$

##### 3.5.2.2. Spatial overlapping deviating patterns.

Finally, we investigated the spatial overlap of abnormal brain maps within groups (Fig. 4.3). First, for each feature, we computed the frequency of abnormality occurrences within each group. Next, the subjects’ abnormal brain maps were transformed into descriptive sets of abnormal features, and the pairwise subject overlap coefficient (OC) and Jaccard similarity (J) were computed within and between groups, Eq. 8, 9. The OC calculates the minimal overlap between two item sets, ranging between 0 and 1, where 1 is totally similar or one set is a subset of the other, Eq. 4. On the other hand, the Jaccard coefficient calculates the total similarity between two item sets, ranging from 0 to 1, where 1 stands for totally similar, thus testing whether two sets share the same members, accounting for all the members, Eq. 7.


(7) $$OC(A,B)=\frac{A\cap B}{min(\;|A|,|B|)},J(A,B)=\frac{A\cap B}{A\cup B}$$

(8) $$Overlap\;within\;group\;:\frac{\sum_{j1}^{N-1}\sum_{j2=j1+1}^{N}X(J1,J2)}{\frac{1}{2}N(N-1)}$$

(9) $$Overlap\;between\;group\;:\frac{\sum_{j}^{N-1}\sum_{m}^{M-1}X(J,M)}{\left(M-1)(N-1\right)},where\;X=0C\;or\;J\;index$$

## 4. Results

### 4.1. Multi-site harmonization effectiveness

We checked the quality of site effect removal performed via M-ComBat application. Before harmonization, all feature set distributions (GMV, WMV, CT) for the HCs among the 8 sites (HCP site and 7 StratiBip sites) resulted significantly different (*p < 1e-29*) but no differences were detected among sites after harmonization (*p > 0.680*).

For BD in the 7 StratiBip sites, all feature sets were significantly different across sites (*p < 1e-12*) before harmonization, whereas statistically significant differences remained for CT and GMV features (*p < 0.018*) after harmonization; the pairwise post-hoc comparisons corrected for multiple tests showed that differences survived for CT features between site 4 and site 6 (Table S3 and Fig. S2). A second quantitative check was performed by probing how the harmonization affected a support vector machine (SVM) model trained to classify sites based on the entire feature set. A substantial decline in average *f1-score* was observed in the validation portion (N=836), from 95 % before harmonization to 23 % after harmonization, and all sites showed a decrease in *f1-score* to below chance-level (Table 1). Group- and feature set-specific SVM site classification results were also extracted (Table S4). Further analyses assessing M-ComBat performances in terms of biological effect preservation were performed (Fig. S3, Tables S5-8).

### 4.2. AE-based normative model performance

When trained on the HCP-YA training set, the AE normative model achieved a training loss MSE of *0.182* (*[0.179;0.185]; 95 % CI*) and a validation loss MSE of *0.222* (*[0.211;0.233]; 95 % CI*) after 2000 training epochs (Fig. S4). After training, we extracted the AE model reconstructions for the StratiBip external test set data and computed the respective REs and MDS by subject, by group, and by feature-by-group. Concerning the subjects’ MDS, as expected, the BD group showed a significantly higher MDS median, *0.2264* (*[0.2210,0.2324]; 95 % CI*) compared to the HC group, 0.1988 (*[0.1945,0.2030]; 95 % CI*). Such difference was statistically significant since the CI for the two groups did not overlap, or, in other terms, the median MDS difference CI did not include zero, *−0.02760* (*[−0.03390, −0.02155]; 95 % CI*). The feature-wise MDS 95 % CIs are reported in Fig.S5.

### 4.3. Group-level BD vs. HC comparisons

#### 4.3.1. BD-deviating brain features

**Fig. 5.**
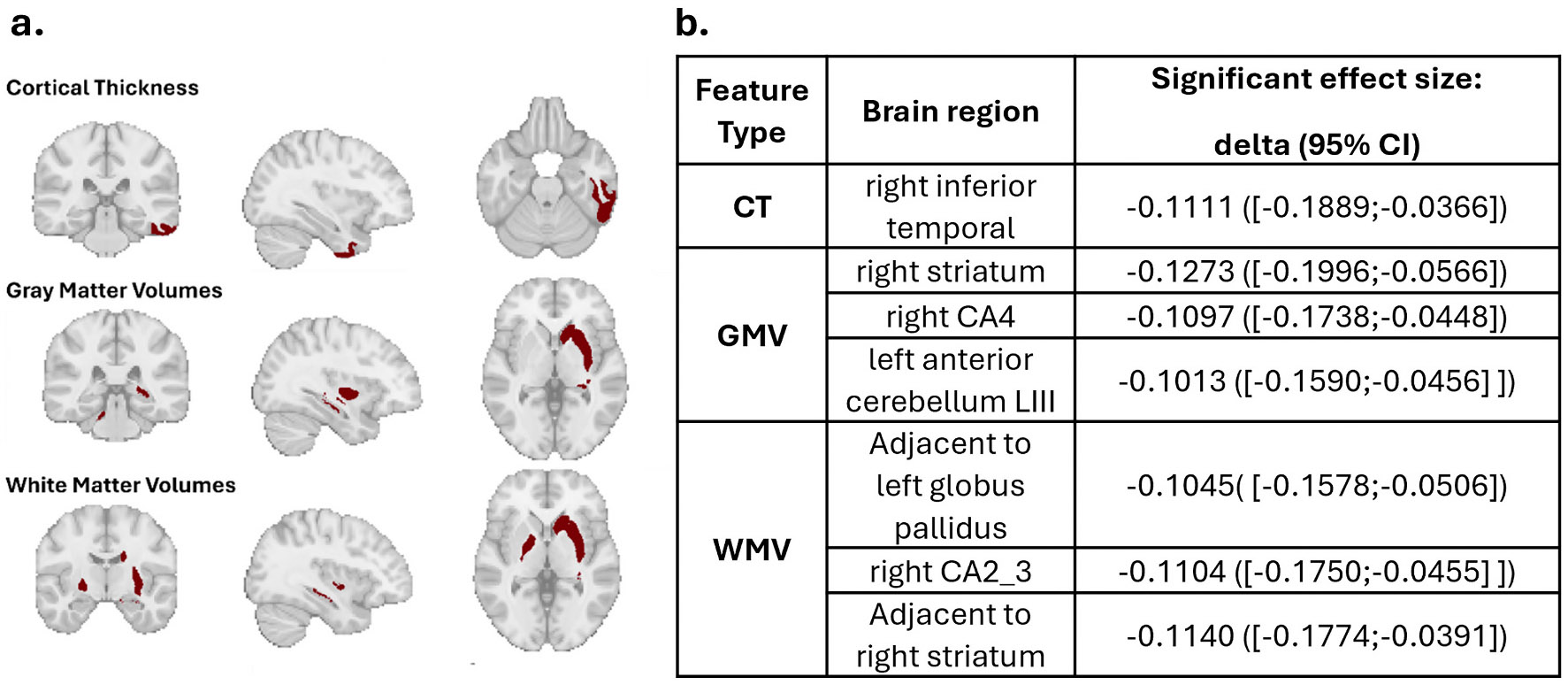
Brain features with significantly higher deviations in BD. a, Representation of all brain features that were identified with significantly higher deviations in the BD group. b, Table describing the identified features and their associated 95 % CI cliff’s delta effect size.

We employed the trained AE model to extract the StratiBip external test set REs and calculated the respective MDS. Several features from all types (CT, GMV, WMV) were found to have significantly higher deviations in the BD group, identified by a significant Cliff’s delta effect size and an uncorrected bootstrap mean estimate *pvalue < 0.05* (Fig. 5). We identified higher BD deviations in CT in the right inferior temporal gyrus, and in volumes of subcortical and adjacent regions belonging to the cerebellum and the limbic system (hippocampus, striatum, globus pallidus). To provide a reference for the AE model findings, we also performed a standard mass-univariate statistical BD vs. HC comparison using a two-tailed MWU test (*p < 0.05; uncorrected and FDR corrected*). Only the WMV surrounding the left globus pallidus emerged as significantly different after correcting for multiple tests (Table S11).

#### 4.3.2. RE patterns heterogeneity

**Fig. 6.**
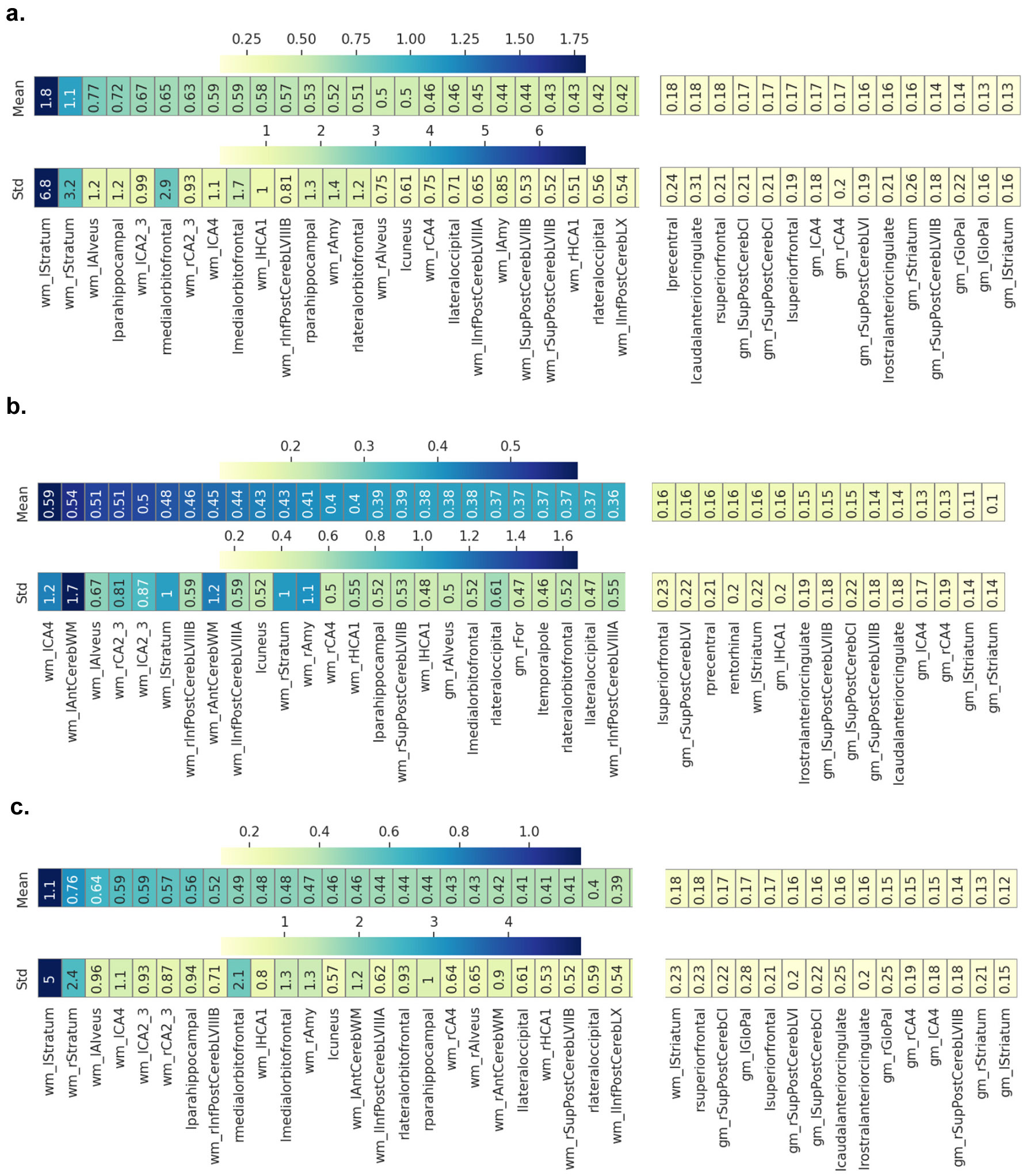
RE heterogeneity within and between groups. The mean and standard deviation RE pairwise differences are shown in a sorted heatmap, including 25 features with the highest heterogeneity levels and the least 15, for a, BD group; b, HC group; c, between the two groups HC vs.BD.

We then quantified the feature-wise RE heterogeneity within and between each group by computing the average RE differences across pairs of subjects (Fig. 6). In general, RE patterns were more homogeneous in the HC group, with a maximum mean pairwise difference and standard deviation of *0.59* ± *1.2*, compared to *1.8* ± *6.8* in the BD group. In Table 2. we summarize the main results and show the highest-ranking features in terms of mean heterogeneity in within-BD, within-HC and between-groups. Overall, for both groups, CT and WMV features presented higher levels of heterogeneity than GMV features. Among all features, the WMV of the left and right Stratum displayed the highest pairwise RE difference among BD subjects, ranking 1st and 2nd in terms of heterogeneity (Fig. 6a and Table 2), but not among HC subjects, ranking 6th and 11th (Fig. 6b); of note, these features showed the highest group difference, i.e., the absolute pairwise difference between subjects’ RE from the two groups, ranking 1st and 2nd (Fig. 6c and Table 2). In the BD group, other features with high mean RE heterogeneity included WMV of the left alveus, left HCA1, left and right CA2_3 and left CA4, and CT of left para-hippocampal gyrus. In addition, the CT of bilateral medial orbitofrontal cortex showed the highest RE standard deviation within-BD and overall, along with the WMV of bilateral Stratum. In the HC group, the WMV of left CA4 and anterior cerebellum displayed the highest heterogeneity, followed by the left alveus, right CA2_3, left CA2_3, and left Stratum. Apart from WMV in the left and right Stratum, the features differing the most in terms of RE magnitudes between HC and BD groups included WMV in left alveus and CA4, bilateral CA2_3 and CT of para-hippocampal gyrus.

#### 4.3.3. RE extreme deviations

**Fig. 7.**
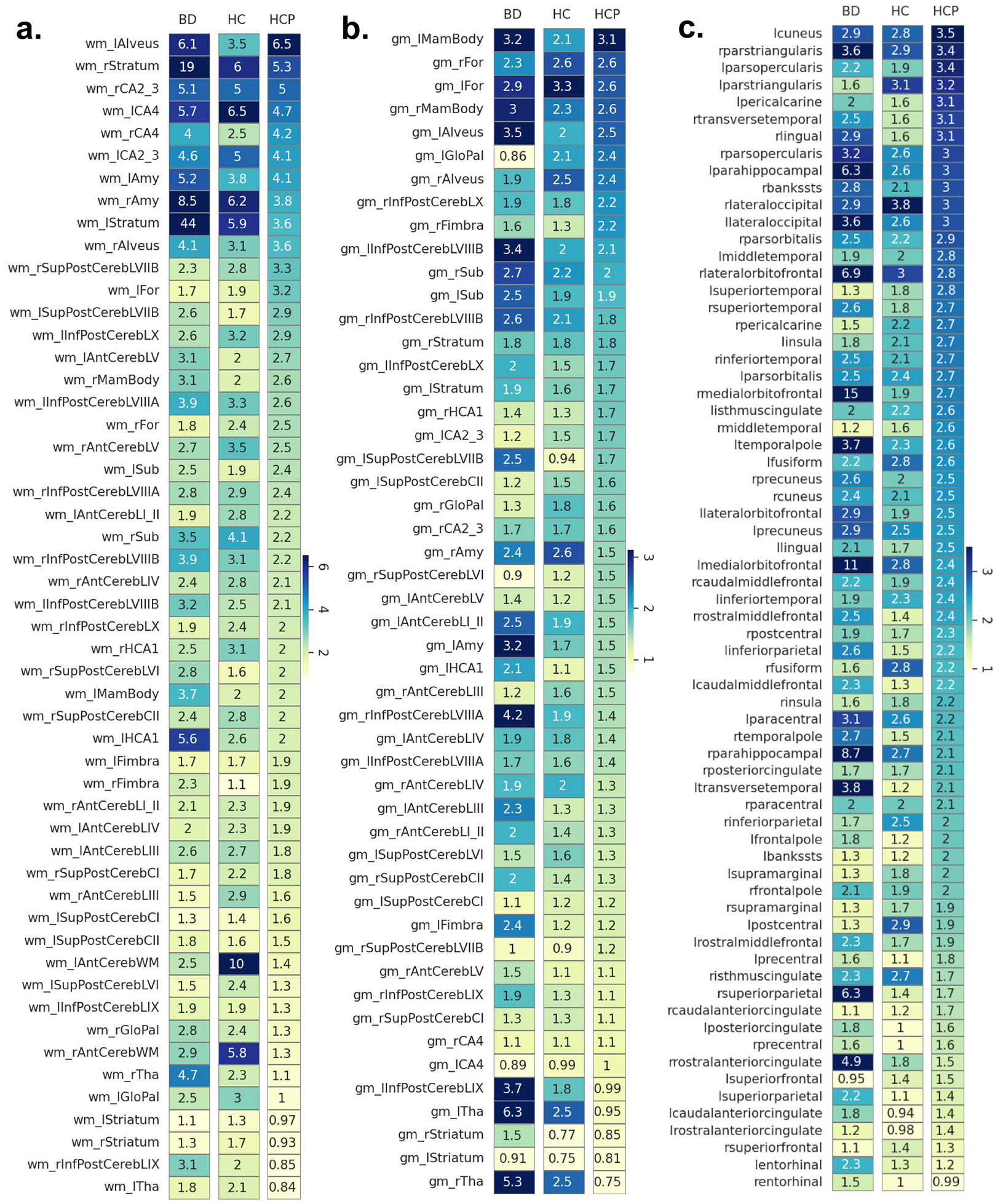
Feature MEVs in the normative HCP-YA and StratiBip BD and HC test set groups. In the top heatmaps (a. WMV features, b. GMV features, c. CT features), the feature-wise MEVs for the StratiBip BD (BD column), StratiBip HC (HC column) and normative HCP-YA (HCP column) groups are plotted. Features are sorted in descending order based on the normative HCP-YA MEVs. The StratiBip BD and HC group heatmaps are color-coded in the same range as the normative HCP-YA one to highlight deviations from the normative expectation within the same brain feature.

We then modelled extreme REs applying a block maxima approach, where each feature was summarized by its extreme values within each group (HC, BD, HCP-YA). Employing a LOO-CV strategy, we retrieved unbiased reconstructions for each subject in the normative HCP-YA training set and constructed a normative RE distribution for each feature. Including only the top 1 % REs (99 % trimmed), we compared the MEV between the normative HCP-YA training set and StratiBip HC and BD test sets (Fig. 7). In WMV and CT feature sets, the overall maximum MEV in the normative group resulted lower when compared with the 2 StratiBip groups; conversely, all GMV features in the StratiBip HC group resulted within the respective normative group range. In all feature sets, selected features showed MEV differences among the three groups. In general, the BD group was characterized by a more pronounced extreme value profile, resulting in 7 CT, 4 GMV, and 4 WMV features with at least a double MEV compared to the normative and the StratiBip HC groups (Table 3). In contrast, in the HC group, only 2 WMV features showed at least a double MEV compared to both the normative range and BD group (Table 3).

#### 4.3.4. BD vs. HC MDS-based discrimination

We assessed whether the subjects’ MDS would enable the discrimination between the BD group and HC one in the StratiBip test set, achieving an AUC-ROC of *0.6129* (*[0.5989, 0.6270]; 95 % CI*). The optimal MDS threshold to differentiate HC vs. BD was *0.2138* (*[0.2096,0.2181]; 95 % CI*) which yielded a mean accuracy of *58.3 %* (*[56.4 %;60.4 %]; 95 % CI*). Then, we inspected whether accounting for extreme value statistics would enhance this discrimination. This time, each subject was summarized by its extreme values under a block maxima approach, with the MEV (99 % trimmed). Then, the ROC curve analysis was repeated, obtaining an AUC-ROC of *0.6218* (*[0.5999, 0.6452]; 95 % CI*), for an optimal MDS threshold to differentiate HC vs. BD of *1.9032* (*[1.8417,1.9723]; 95 % CI)* yielding a mean accuracy of *59.0 % ([56.2 %;61.8 %]; 95 % CI*), a slight improvement when compared to using central tendency statistics to summarize the RE outcomes, i.e., the MDS.

### 4.4. Personalized brain deviating maps

**Fig. 8.**
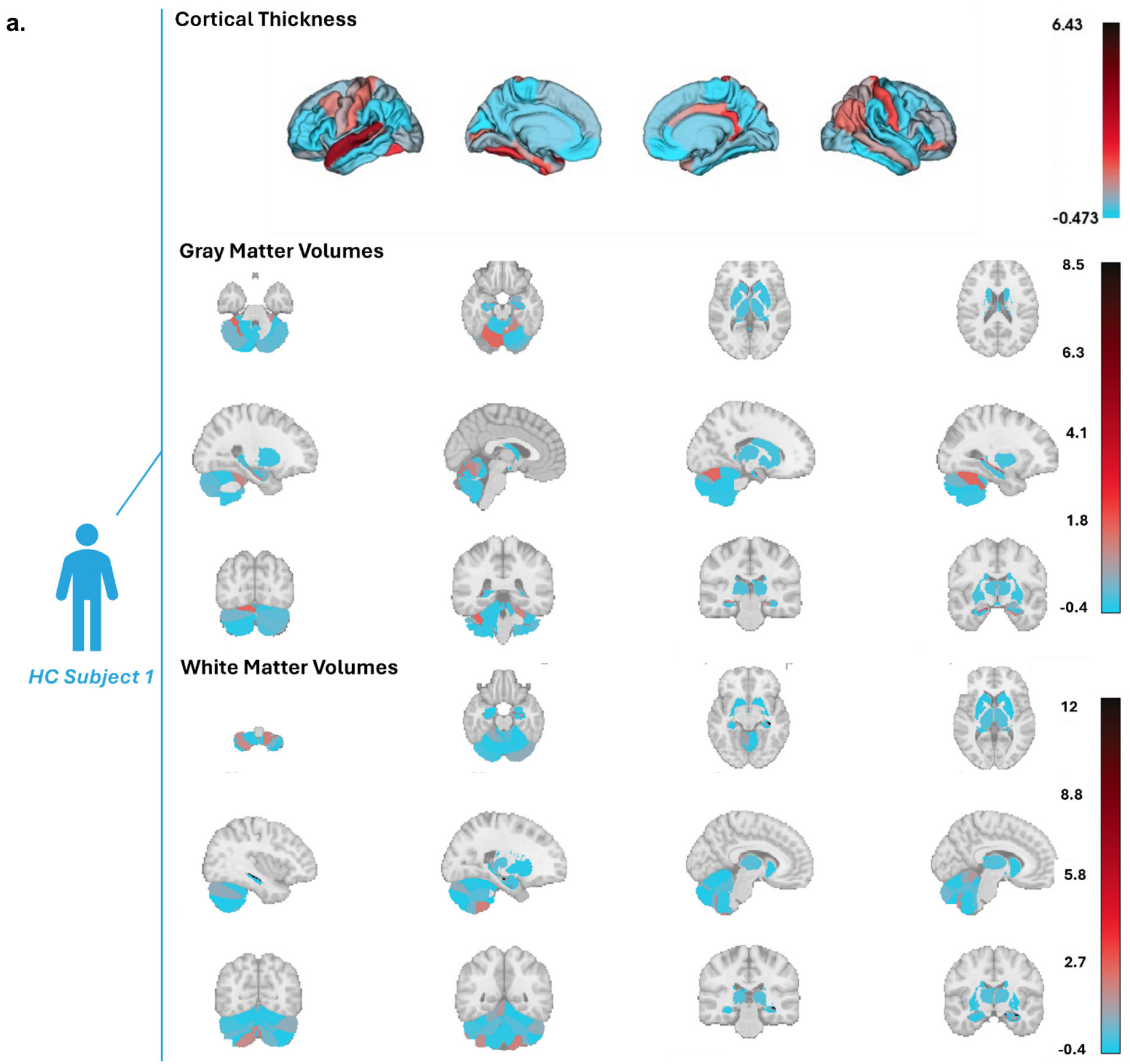

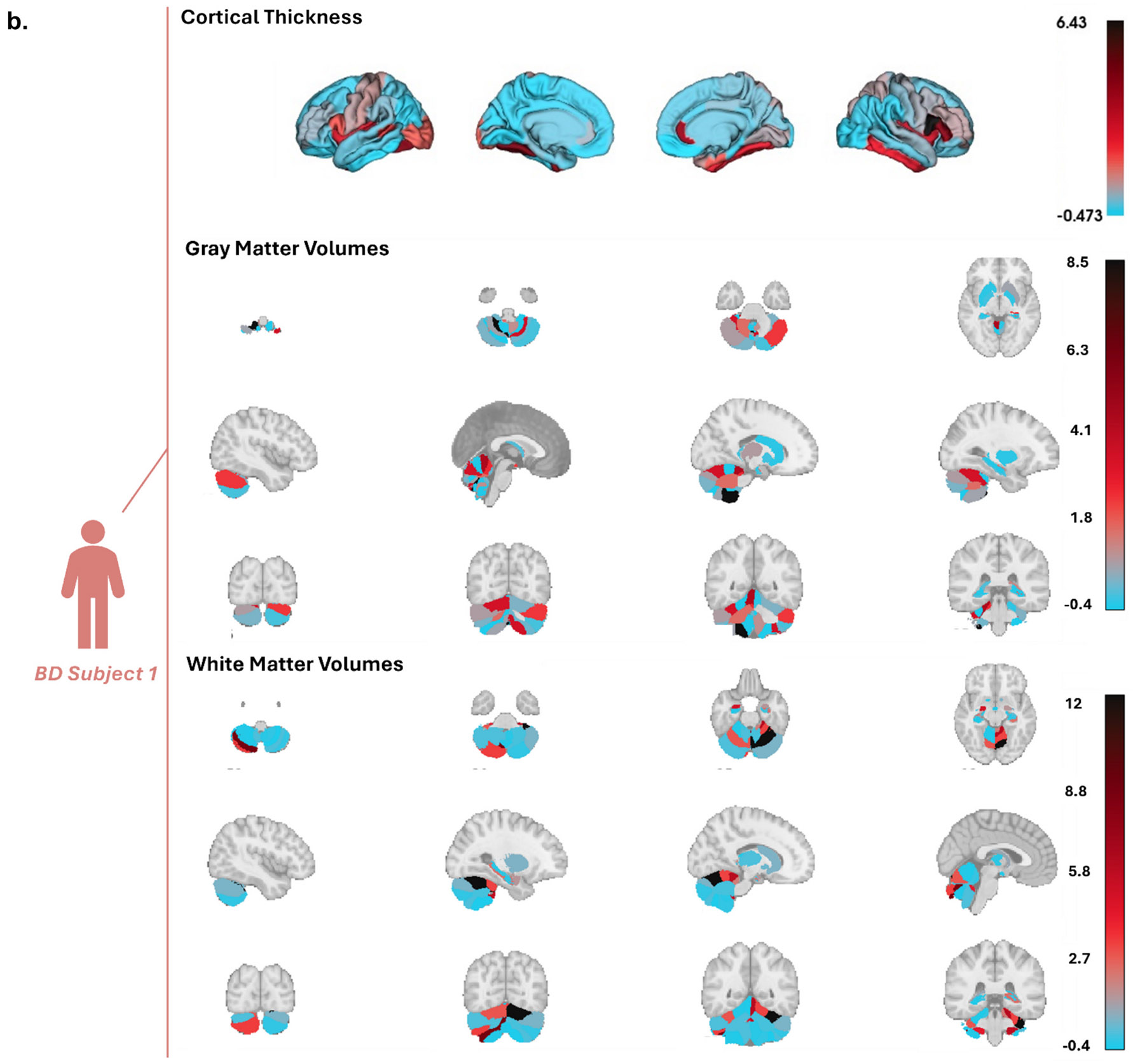
Individual deviating brain maps. We plot the deviating CT, GMV, and WMV feature maps for 2 subjects: a, HC subject CT, GMV and WMV mZ scores; b, BD subject CT, GMV and WMV mZ score. The color bar range is shared between the two subjects for each feature set group to better highlight differences in the mZ scores in the deviating maps.

Individual brain deviations were also employed for subject-level statistical inference. We calculated the mZ for the StratiBip dataset using the HCP-YA feature-wise median and MAD. Then, for each feature, we retrieved the 99th percentile in the normative HCP-YA distribution and used it as the normative mZ threshold, enabling the identification of subject-level abnormal features (*mZ > 99th percentile*) for each StratiBip individual (Fig. S6). We report the resulting brain CT, GMW, and WMV deviating maps of two exemplar subjects from the StratiBip test set, one control and one with BD (Fig. 8). The mZ distributions of all features in the StratiBip HC and BD groups are reported in Fig. S7.

**Fig. 9.**
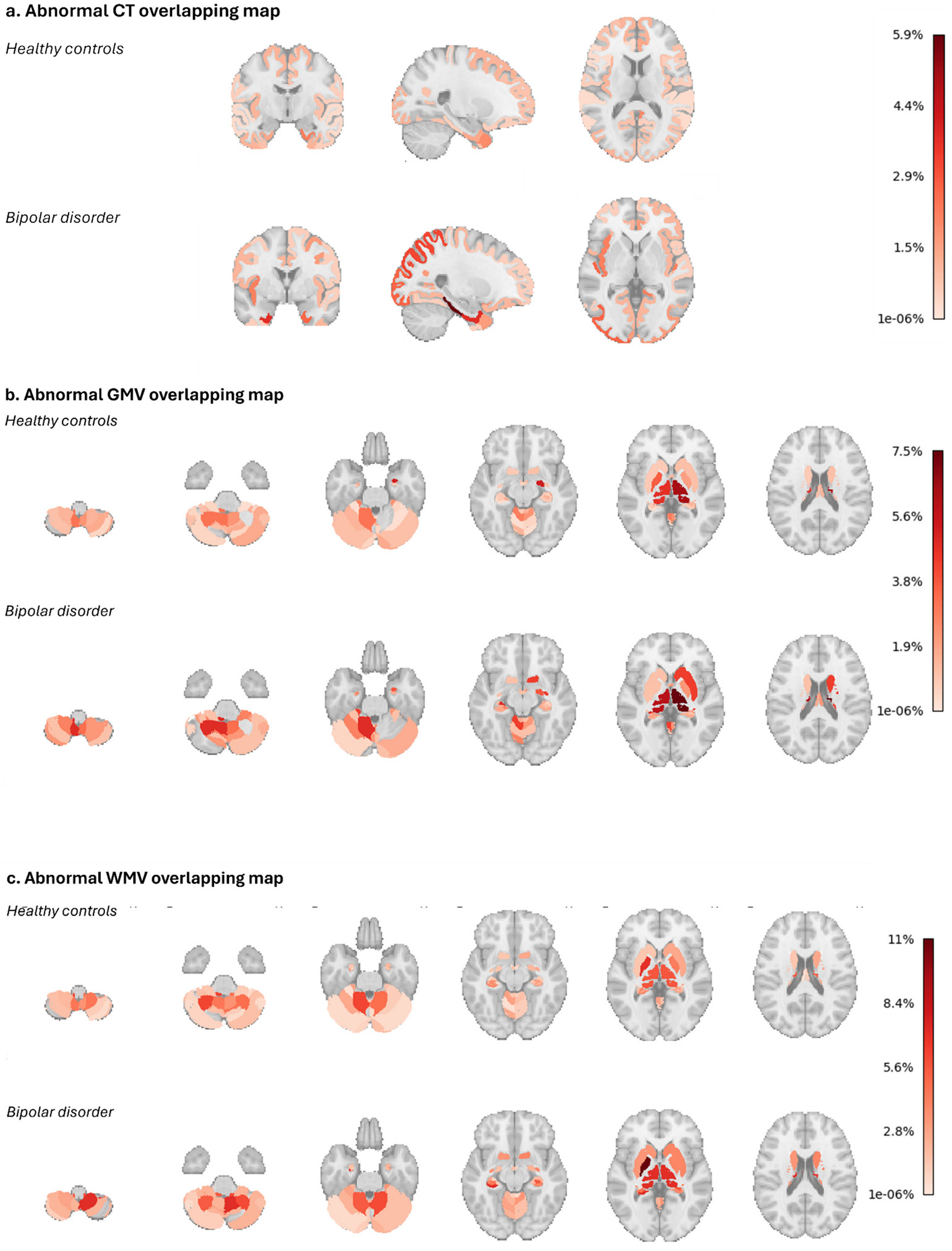
Feature set percentage of abnormalities for each group. The brain maps show the prevalence of individuals, in percentage, with identified abnormalities in each feature within each group, HC and BD.

Next, for the two StratiBip groups, we inspected the prevalence of subject-level abnormal features. Across all feature sets, subjects belonging to the HC group had an average of *1.3 %* abnormal features, corresponding to about *2* features per subject, while in the BD group, this average percentage increased to *1.9 %*, corresponding to *3* abnormal features per subject. For each feature, we inspected the percentage of abnormal occurrences for each group (Fig. 9) and summarized the top-ranked features in Table 5. In the BD group, the highest prevalence (*11 %* of subjects) was found for the WMV adjacent to the left globus pallidus, followed by the GMV of the right thalamus (*7.5 %*) and WMV: of right inferior posterior CerebLIX, surrounding the bilateral thalamus, of left HCA1 and right inferior posterior CerebLVIIIB (*7 %*). Of note, in the HC group, the highest frequency of abnormal cases was also observed for the WMV adjacent to the globus pallidus (*6.9 %*), followed by GMV of right thalamus (*6.3 %*), WMV of left anterior Cerebellum (*6.1 %*) and adjacent to bilateral thalamus (*5.5 %*), and GMV of right amygdala (*5.2 %*). The intra-group and inter-group similarity was also assessed by employing the average pairwise overlap coefficient (OC) and the Jaccard similarity index (J), achieving (i) in the HC group, higher level of similarity compared to the BD group (*OC*_*HC*_ = *0.72; OC*_*BD*_ = *0.60* ∣ *JC*_*HC*_ = *0.32; JC_*BD*_ = 0.23)*, (ii) in the BD group, lower level of similarity compared to the inter-group one (BD-HC) (*OC*_*BD*_ = *0.60; OC*_*HCvs.BD*_ = *0.67* ∣ *JC_*BD*_ = 0.23; JC_HCvs.BD_ = 0.27*).

## 5. Discussion

In this study, we designed a generalizable, and extendable end-to-end pipeline for brain morphological multivariate normative modelling and personalized anomaly detection based on deep AEs. Although AEs have been previously proposed in literature for brain normative modelling [23], our approach is distinct due to the innovative inclusion of a generalizable CR step that enabled the effective translation of our model to external datasets. We leveraged this unique feature by testing our model with a multi-site external test set, following successful harmonization with the training set. Another innovative aspect is the integration of multimodal data, facilitated by the multivariate nature of AEs [62-64]. In the search for brain morphological alterations in BD, our study is the first to employ a multivariate normative framework that integrated CT, GMV, and WMV features for the subject- and group-level characterization of this complex disease.

The AE-based normative model was developed on brain regional features from the large normative HCP-YA cohort and evaluated on features from the external multi-site StratiBip cohort, including both controls and individuals with BD.

First, we showed the effectiveness of the proposed CR pipeline in removing site-related effects from the external multi-site StratiBip test set. This allowed us to integrate datasets acquired in different sites, enabling robust comparisons and increasing statistical power in the test set. Then, we demonstrated the effectiveness of our approach in characterizing brain morphological deviations, identifying subject- and group-level tendencies, as well as heterogeneity and extreme deviations within and between groups.

Our findings indicated that, on average, group-level deviations were higher in BD compared to HC; in the BD group, RE patterns were also more heterogeneous and with greater extreme values than in the HC group. At the individual level, the most prevalent abnormal features were similarly observed in both groups, but prevalence was consistently increased in BD. Notably, we also found greater spatial overlap in individual-level brain abnormal maps between BD and HC subjects, than within the BD group itself.

The latter evidence is in line with the hypothesis that brain morphological alterations in BD, and in general in psychiatric disorders, are subtle and might be nested within the spectrum of normative interindividual variability. In support of this hypothesis, our study did not identify a brain morphological marker for BD as a whole; the group-level deviations were not replicable at the individual level except in a small percentage of subjects. These findings support the conceptualization of BD as a non-unitary disease, exhibiting a variety of neurobiological dimensions, whose characterization paves the way to the identification of personalized signatures of disease and more effective interventions.

### 5.1. CR pipeline was effectively applied to external datasets

In this study, we presented a novel CR framework that enabled the generalization of our DL normative model to external datasets. To the best of our knowledge, this is the first study to demonstrate effective mitigation of site-related and biological confounding effects in a DL analysis pipeline in an EV framework. We considered working with confounder-free data as a prerequisite towards more interpretable DL models. The inability to ascertain the information that drives the performance of a ML model can lead to erroneous result interpretations, known as the source ambiguity problem [26,53,65]. To address this challenge, it is recommended to implement a strategy of controlling for alternative sources of information from the target of interest, a process referred to as covariate adjustment or confounding-effects correction. In S. Rutherford et al. [66] a methodology was put forward for expanding a pretrained Bayesian regression model to data from novel sites. However, site-related variation is modelled with features-of-interest in a single regression model including the site variable as covariate, impending its usage in our deep learning framework. M-ComBat was shown to effectively harmonize data from different sites and has been recently employed in a multi-site PET study in an external validation framework [50]. In this study, we applied the M-ComBat strategy in a normative framework and demonstrated its efficacy in harmonizing external StratiBip test sets with the HCP-YA training set. In both harmonization and biocovariates models, the normative site and biological confounding effects were assumed to be generalizable to patient data and any associations between diagnosis and brain features were not modelled, as this could have led to data leakage problems and consequently biased the model results [53,54].

### 5.2. AE-based normative modelling empowered the identification of group-level brain morphologic deviations in BD

Group-level analyses on brain morphological correlates of psychiatric disorders have been extensively performed in literature, but only a few in terms of normative deviation metrics [14,20,22,23,67]. Since normative models can detect individual deviations from the norm, they are especially suitable for unravelling brain heterogeneity in BD. Our findings showed higher median deviations in BD compared to HC; specifically, volumes of the basal ganglia and adjacent to it (striatum and globus pallidus) and from the hippocampus (CA4, CA2_3) revealed increased deviations in BD compared to HC. The WMV surrounding the globus pallidus was also significant in the mass-univariate case-control analysis that we used as reference, supporting the neurobiological plausibility of the AE-based normative findings. These group-level deviations are in line with existing literature on BD, suggesting morphological alterations in brain regions involved in affective processing, including the basal ganglia, hippocampus, and temporal regions observed in our study. In the case-control mega-analyses of the ENIGMA BD Working Group, BD was found to be associated with cortical thinning in inferior temporal regions and with volumetric reduction in the hippocampus [4,10]. Additionally, in another study employing a univariate normative approach, individuals with BD were also reported to have GMV deviations in cerebellar and temporal regions [14].

While the overall agreement with the existing evidence supports the reliability of the deviations observed in our BD sample, it should be considered that our multivariate findings reflect patterns of alterations rather than region-specific changes.

Regarding the BD group discrimination, the whole-brain MDS presented a low discriminative power when compared with the state-of-art, achieving an AUC-ROC of 0.61 and an accuracy of 58.3 % using the best MDS threshold. A recent review on ML studies that attempted to classify BD vs. HC reported a range of prediction accuracies between 59 %–78 % based on WMV and GMV predictors [68] in parallel, the ENIGMA BD Working Group reported an AUC-ROC of 0.7149 (0.6939–0.7359) using cortical thickness, surface area, and subcortical volumes; this improved performance could be due to different factors, like the inclusion of a bigger BD sample or the non-removal of biological effects from the brain features used for classification [69].

### 5.3. Distribution and extreme values analyses highlighted brain morphology heterogeneity in BD

Our normative model was exploited to assess and compare the heterogeneity and extreme profiles of the deviating patterns in BD and HC groups.

BD individuals presented higher levels of heterogeneity, especially for WMV in subfields of the hippocampus, alveus, and cerebellum, and for CT of parahippocampal and medial orbitofrontal regions. The highest difference between groups, highlighting much greater heterogeneity in BD, was found for the WMV of the bilateral stratum. This more marked heterogeneity of REs reflects a greater model variability in reconstructing the data, which in turn is suggestive of brain morphological heterogeneity in the BD group.

Interesting evidence on BD was also provided by the assessment of extreme values; Our findings suggest more pronounced extreme deviations in BD, being characterized by the greatest number of features with a MEV that was more than the double of both StratiBip HC and HCP-YA groups. Moreover, the discrimination between the BD and HC groups improved when using MEV scores instead of MDS as subject-level deviating scores, achieving an AUC-ROC of 0.62. This suggests that examining extreme values can enhance the separability between groups.

High extreme deviations were found in features that showed marked heterogeneity in the BD group, including WMV of bilateral stratum and left HCA1 and CT of left parahippocampal and bilateral medial orbitofrontal regions. We hypothesize that the heterogeneity was driven by the incidence of extreme values in these features, possibly reflecting pronounced phenotypic differences in BD.

Notably, in *Li Z.* et al. [30] authors identified normative deviation scores of the GMV on the left middle orbital frontal gyrus as the most reproducible feature to discriminate BD from HC, applying a random forest classifier. We hypothesize that only a sub-group of subjects present more severe alterations in these regions, and this may drive both the increased heterogeneity/extreme values observed in the present results and the higher discriminatory stability in the second. The enhanced brain heterogeneity could underlie the phenotypic variability of individuals affected by BD, which has impeded so far the identification of objective brain markers of disease [70].

### 5.4. AE-based normative modelling empowered the creation of personalized brain deviating maps

The individual brain deviation maps were constructed with the mZ scores and a conservative 99th percentile threshold was used as abnormality criteria to binarizie the deviating maps.

On average, subjects affected with BD and HCs showed a similar percentage of abnormalities, slightly higher in BD (1.9 %) than in HC (1.3 %). The maximum spatial overlap of features identified as abnormal was identified for the WMV surrounding the globus pallidus, expressed in 11 % of BD subjects and in 6.9 % of HCs, followed by the GMV of the right thalamus (7.5 % in BD, 6.3 % in HC). Interestingly, previous univariate normative studies on BD reported the highest spatial overlap of abnormalities in the thalamic region, showing around 2 % in [14], and 5.17 %–8.19 % in [71], and high discriminatory stability of GMV thalamus deviations [30]. Overall, our results show that abnormalities in BD spread mostly through the volumes of the bilateral thalamus and adjacent to it, hippocampus subregions, and cerebellum.

Of note, this personalized inference on BD subjects unravelled brain morphological abnormalities in regions that did not emerge from the group-level comparisons. These regions included the thalamus, for which volumetric alterations have been previously reported in case-control mass-univariate comparisons [4]. It should be noted that thalamic volume was deviating in a number of HC and BD subjects, albeit with higher frequency in the last group. This might be attributed to thalamic alterations being nested in healthy variations, overcoming the expected normative variability only for a subset of subjects with BD.

Overall, across all features, we found a lower overlap of individual abnormalities in BD than in HC. In the BD group, the pairwise abnormal spatial maps comparisons showed that a minimal subset of abnormal features replicated on average (OC = 60 %), but the complete spatial overlap was lower (J = 23 %). Noticeably, abnormal profiles of BD subjects overlapped more with other HCs than with other BD subjects. These results further asserted the heterogeneity of BD and are in agreement with the accumulating evidence that brain changes in BD, as in other psychiatric disorders, might be nested within healthy variations [20,72].

### 5.5. Our normative framework included key innovations that improve its translational relevance

We advanced the translational aspects of our model with two main innovative implementations: (1) the development of a generalizable confounder removal pipeline and, (2) the individual-level assessment of brain normative deviations. The ability to generalize the proposed end-to-end pipeline to external datasets represents a critical advancement, addressing significant challenges related to the distribution and practical use of software in clinical practice, paving the way for broader applicability in real-world clinical settings. Then, the brain individual-level assessment enabled by our multivariate normative modelling proposal constitutes a promising avenue for translating research into clinical practice. We identified overlapping brain-deviation patterns across subjects in HCs and BD groups, which could be extended to multi-disorder cohorts and, with sufficient data, be used to stratify and subtype patients, independently of formal psychiatric diagnoses. An increasing body of evidence is remarking the need to adopt a dimensional perspective for identifying the brain endophenotypes of clinical dimensions that are shared between BD and other disorders in the psychotic or affective spectrum [73,74]. This tool has the potential to facilitate the implementation of personalized disease models in clinical practice, and guide more tailored treatment strategies, ultimately improving therapeutic outcomes and advancing precision psychiatry.

## 6. Limitations

The implemented methodology has several limitations regarding the adjustment of biological covariates. For instance, the data was not corrected for medication on BD, therefore we cannot exclude the possibility that the significant group differences and brain deviations may be driven by medication effects. Similarly, we did not account for comorbidities which might be important to distinguish between disorder-specific effects and others. While the utilization of confounder-free data contributes to the development of more interpretable DL models, ComBat and biological covariates linear regression are subject to limitations concerning confounding source modelling. The former relies on a Bayesian framework for statistical inference of site effects, and the estimates may be affected by sample size and imbalances between sites. Second, linear regression, despite its simplicity and ease of implementation, might not fully capture the biological effects if these encompass non-linearities. To date, there is no gold standard to address confounding effects in neuroimaging studies, and both methods are widely employed in literature.

On another note, the uncertainty of estimation of the MRI-based features has not been thoroughly evaluated. CAT12 brain tissue segmentation is based on algorithms that may encounter challenges in segmenting small brain regions with mixed tissues (gray and white matter) and borders. For example, subcortical gray matter regions within the basal ganglia and thalamus exhibit reduced GM-WM contrast, attributable to their high content of cellular iron, rendering the T1-w signal similar to that of WM [75]. Given the higher probability of erroneous tissue segmentation in these regions, we have opted to incorporate all volumetric estimates derived from CAT12 based on the CoBra atlas. This approach included the estimations of both WMV for GM regions, and vice-versa, interpreted as the volume adjacent to the respective region. These CAT12 estimates may stem from intrinsic limitations in the segmentation software’s voxel-based tissue classification or poor subject-atlas alignment. By utilizing all volume estimates, we avoid excluding potentially relevant information due to cherry-picking selection. Nevertheless, it is important to acknowledge that our results may reflect the inherent uncertainty associated with these volumetric estimations.

Then, our AE-based network exhibits several limitations that warrant consideration. First, as a black-box model, it is challenging to ascertain precisely what the model is learning. This limitation is only partially mitigated by locally tracking the RE, and by the confounder removal pipeline. Second, the RE lacks information about directionality, making it impossible to determine whether a given deviation results from a feature being too low or too high relative to the encoded normative range. This limitation affects potential clinical applications where understanding the direction of brain alterations is crucial. Another significant limitation stems from the difficulty of evaluating false-positive deviations within this unsupervised framework. The absence of a ground truth for brain deviations limits the ability to distinguish true abnormalities from spurious findings—an essential factor for reliable clinical deployment. Consequently, interpreting the brain deviation results warrants caution, and we have focused on broader overall trends, rather than attempting to pinpoint specific brain markers. Abnormal features were identified in both the HC and BD groups, suggesting that encoding normative levels is not straightforward and that true normative ranges may generalize to non-normative data. The larger brain deviations observed in BD did not demonstrate sufficient discriminative power from a clinical applications perspective. Then, from the dataset perspective, there was an incomplete assessment of BD across the entire lifespan, as the dataset only included young adults. Besides, in the StratiBip test set, the sample size and biological covariates were not equally distributed among sites, which could have affected the results. To create a more generalizable framework, future works should focus on increasing dataset diversity and numerosity.

With regard to the brain features, ROI-based analysis was employed instead of utilizing all voxel-based whole-brain data. While voxel-based deviations would have offered increased granularity for detecting brain abnormalities, this approach does not come without its challenges. The significant increase in dimensionality could introduce greater noise into the model, leading to overfitting, potentially degrading its performance and reducing model’s generalizability. This issue could also adversely affect subsequent stages of the pipeline, potentially exacerbating false positive rates—an evaluation we were unable to conduct in this study as mentioned previously. ROIs defined based on extensively validated brain atlases allowed us to retain whole-brain data information while clustering voxels in a morphologically and anatomically relevant way, contributing to great interpretability and effective dimensionality reduction. A natural extension of this work would be to conduct a comprehensive clinical characterization and further explore patient stratification using voxel-based data, by appropriately adapting the deep learning model to accommodate the increased dimensionality.

Lastly, a major challenge for a potential clinical application is that our approach does not directly address the issue of understanding the underlying aetiology of psychiatric disorders. Instead, it operates within an unsupervised framework that assumes and accepts this “unknown” aetiology. The clinical applicability of any medical device, including diagnostic software or predictive models, may be limited in the absence of causal disease models. In future developments, the pipeline should be expanded to map individual brain deviations to relevant clinical dimensions (e.g., those relative to treatment, behavioural profiles, environmental risk factors) that might facilitate not only the understanding of brain morphological variability but also the translation of knowledge of individual brain deviations in the psychiatric practice. The proposed framework has the potential to serve as a foundational tool for developing personalized disease models in future clinical settings, particularly through the ongoing accumulation of data and outcomes over time.

## 7. Conclusion

In this study, we developed a generalizable end-to-end multivariate normative modelling and anomaly detection framework based on deep AEs. The novelty of our pipeline resides in the integration of data harmonization and biological confounder removal, with normative modelling in an external validation framework, which significantly improved the translational relevance of our model. We demonstrated the successful application of this framework in the search for brain morphological deviations in BD. This was achieved by integrating CT, GMV and WMV with a normative AE model trained with the HCP-YA cohort, leveraging anomaly detection on an external multi-site test set composed of HC and BD. Our findings support the hypothesis that brain morphological alterations in BD are heterogeneous and partly nested within healthy interindividual variations, remarking the importance of moving from categorical diagnoses to a transdiagnostic dimensional perspective. In this perspective shift, our multivariate normative modelling framework could capture individual brain differences that might be used for making more effective and personalized clinical decisions.

## Supplementary Material

1

## Figures and Tables

**Table 1:** F1-score SVM site classification before and after harmonization.

	HCP	StratiBip							All
			
F1-score	HCP-YA (*N* = 555)	1 (*N* = 38)	2 (*N* = 82)	3 (*N* = 51)	4 (*N* = 14)	5 (*N* = 41)	6 (*N* = 33)	7 (*N* = 22)	weighted average (*N* = 836)
Before Harmonization	1.00	0.88	0.89	0.94	0.38	0.99	0.72	0.67	0.95
After Harmonization	0.30	0.03	0.08	0.12	0.00	0.15	0.12	0.09	0.23

**Table 2:** Features with higher mean heterogeneity RE patterns ranked by group. The first sub-table, BD Heterogeneity rank, concerns the BD rank and orders the columns in descending fashion (higher heterogeneity to lowest) and the respective HC-group and inter-group results were added for the sake of comparison. The second sub-table, HC Heterogeneity rank, orders the columns with the HC rank in the same manner. Highest values for each column and sub-table are highlighted in bold.

	1^st^	2^nd^	3^rd^	4^th^	5^th^
BD Heterogeneity rank	L-Stratum (WMV)	R-Stratum (WMV)	L-Alveus (WMV)	L-parahippocampal (CT)	L-CA2_3 (WMV)
**BD-StratiBip**	**1.80 ± 6.80**	**1.10 ± 3.10**	**0.77 ± 1.20**	**0.72 ± 1.20**	**0.67 ± 0.99**
HC-StratiBip	0.48 ± 1.00	0.43 ± 1.00	0.51 ± 0.67	0.39 ± 0.52	0.50 ± 0.87
Inter-Group	1.10 ± 5.50	0.76 ± 2.40	0.64 ± 0.96	0.96 ± 0.94	0.59 ± 0.93
**HC Heterogeneity rank**	**L-CA4** **(WMV)**	**L-AntCerebWM** **(WMV)**	**L-Alveus** **(WMV)**	**R-CA2_3** **(CT)**	**L-CA2_3** **(WMV)**
**HC-StratiBip**	0.59 ± 1.20	**0.54 ± 1.70**	0.51 ± 0.67	0.51 ± 0.81	0.50 ± 0.87
BD-StratiBip	0.59 ± 1.10	0.38 ± 0.46	**0.77 ± 1.20**	**0.63 ± 0.93**	**0.67 ± 0.99**
Inter-Group	0.59 ± 1.10	1.20 ± 0.46	0.64 ± 0.96	0.57 ± 0.87	0.59 ± 0.93

L: left hemisphere; R: right hemisphere; W/GMV: white/gray matter volume; CT: cortical thickness.

**Table 3:** Summary of features with at least a double MEV (highlighed in bold) compared to the other groups.

Features		BD- StratiBip	HC- StratiBip	HCP- YA
BD group vs. others				
CT	Left parahippocampal gyrus	**6.9**	3.0	3.0
	Right parahippocampal gyrus	**8.7**	2.7	2.1
	Right lateral orbitofrontal	**6.9**	3.0	2.8
	Left medial orbitofrontal	**11.0**	2.8	2.4
	Right medial orbitofrontal	**15.0**	1.9	2.7
	Right superior parietal	**6.3**	1.4	1.7
	Right rostral anterior cingulate	**4.9**	1.8	1.5
GMV	Right Inferior posterior	**4.2**	1.9	1.2
	CerebLVIIIA			
	Adjacent to left Fimbra	**2.4**	1.2	1.2
	Left Thalamus	**6.3**	2.5	1.2
	Right Thalamus	**5.3**	2.5	0.75
WMV	Right Stratum	**19.0**	6.0	5.3
	Left Stratum	**44.0**	5.9	3.6
	Left HCA1	**5.6**	2.6	2.0
	Adjacent to right Thalamus	**4.7**	2.3	1.0
HC group vs. others				
WMV	Left Cerebellum	2.5	**10.0**	1.4
	Right Cerebellum	2.9	**5.8**	1.3

**Table 5:** Summary of abnormal features spatial overlap ranked by group. The first sub-table concerns the BD rank and orders the columns in descending fashion (higher spatial overlap to lowest) and a row with the respective HC-group results is added for the sake of comparison. The second sub-table orders the columns with the HC rank in the same manner.

	1^st^	2^nd^	3^rd^	4^th^	5^th^	6^th^
**BD rank**	L-Globus Pallidus (adjWMV)	R- Thalamus (GMV)	R-InfPostCerebLIX (WMV)	R- Thalamus (adjWMV)	L- Thalamus (adjWMV)	L- HCA1 (WMV)
**BD-StratiBip**	**11.0 %**	**7.5 %**	**7.0 %**	**7.0 %**	**7.0 %**	**7.0 %**
HC-StratiBip	6.9 %	6.3 %	3.3 %	5.5 %	5.5 %	3.6 %
**HC rank**	L-Globus Pallidus (adjWMV)	R- Thalamus (GMV)	L- AntCerebWM (WMV)	R- Thalamus (adjWMV)	L- Thalamus (adjWMV)	R- Amidgala (GMV)
**HC-StratiBip**	**6.9 %**	**6.3 %**	**6.1 %**	**5.5 %**	**5.5 %**	**5.2 %**
BD-StratiBip	11.0 %	7.5 %	5.3 %	7.0 %	7.0 %	3.7 %

adjWMV: adjacent WMV; L: left-hemisphere; R: right-hemisphere.

## Data Availability

The HCP-YA normative dataset is publicly available on connectomeDB platform (https://db.humanconnectome.org). The StratiBip dataset is governed by data-use agreements or sponsor restrictions and therefore not publicly available.

## Code availability

The custom code used in this study is available for research purposes (GitHub repository https://github.com/inesws/Normative_AE.git). A demo test code is available that allows to try the trained model with some pre-processed and corrected HCP-YA exemplar data. In order to apply the trained model to new data, researchers should follow the instructions.

## Appendix A. Supplementary data

Supplementary data to this article can be found online at https://doi.org/10.1016/j.artmed.2024.103063.
